# From pixels to breeding values: genetic analysis of detection and movement traits in laying hens using automated tracking data of ArUco-marked birds

**DOI:** 10.1186/s12711-026-01043-y

**Published:** 2026-04-10

**Authors:** T. Osorio-Gallardo, A. van Putten, D. Janssen, M. F. Schrauf, M. F. Giersberg, T. B. Rodenburg, P. Bijma

**Affiliations:** 1 Wageningen University and Research, Droevendaalsesteeg 4, 6708 PB Wageningen, The Netherlands; 2 Utrecht University, Heidelberglaan 8, 3584 CS Utrecht, The Netherlands; 3 Hendrix Genetics, Spoorstraat 69, 5831 CK Boxmeer, The Netherlands

## Abstract

**Background:**

Automated tracking technologies make it possible to perform large-scale genetic analyses of behavioural traits in livestock. However, behavioural phenotypes derived from automated systems can be noisy, incomplete, or difficult to interpret in terms of specific behaviours or welfare outcomes. This study evaluated the feasibility of using ArUco marker-based tracking to derive three behavioural phenotypes expressed in the litter area (detected or not, minutes detected, walking speed) in a population of 1,132 crossbred laying hens kept under semi-commercial conditions.

**Results:**

Despite the fact that 63% of the time, individuals were not detected due to occlusion or system limitations, the tracking method yielded sufficient data (on average 5,326 detections per individual per hour) to estimate genetic parameters with genomic prediction. All traits exhibited significant additive genetic variance, with heritability estimates ranging from 0.05 to 0.08 for hourly measurements and from 0.13 to 0.26 for daily measurements. Genetic correlations revealed shared architecture between detection traits (detected or not and minutes detected, ${r}_{g}$= 0.71), but an unexpected strong negative correlation between walking speed and minutes detected (${r}_{g}$= −0.73) probably because faster individuals sooner disappeared from the camera’s field of view. Cross-validation accuracies were modest (0.25–0.32), while model-based accuracies were nearly twice as high. Multi-trait modelling did not improve the accuracy of estimated breeding values.

**Conclusions:**

Our results demonstrate that automated tracking can generate useful phenotypes for the genetic evaluation of complex behaviours. The current methodology provides individual-level behavioural data suitable for genetic analyses, though its application at scale still requires substantial effort in data collection and processing. Additionally, further ethological understanding is required to confirm which specific behaviours these traits reflect.

**Supplementary Information:**

The online version contains supplementary material available at 10.1186/s12711-026-01043-y.

## Background

Growing societal concern for animal welfare, as evidenced by recent surveys showing strong public support for welfare improvements, highlights the importance of ethical livestock production [1]. Genetic selection on behavioural traits that can be used as welfare indicators has been proposed as a promising approach to enhance animal welfare [2, 3]. Welfare indicators are behaviours that can be related to positive or negative aspects of welfare. For example, in poultry, foraging and exploration are behaviours commonly associated with positive welfare [4] while feather pecking and piling are associated with negative welfare compromise. Including behavioural traits in breeding programs is not only important for improving welfare but also because social dynamics can influence other traits [5]. Studies have shown that ignoring social behaviours can lead to negative outcomes, such as increased aggression and mortality, while incorporating them can reduce harmful behaviours and promote positive interactions [6, 7]. Additionally, the genetic analysis of behaviour can also help manage correlated responses in other traits. For instance, selecting against aggressiveness may not only reduce harmful social interactions but could also lower feed intake, yielding benefits for both animal welfare and production efficiency [8]. However, balanced selection indexes often dilute the impact on behavioural traits due to competing emphasis on production traits [9], which can be a real constraint for genetic improvement of behavioural traits.

However, the genetic improvement of animal welfare through selection has been fundamentally hindered by the longstanding difficulty in collecting behavioural phenotypes at a sufficiently large scale. Traditional phenotyping methods such as manual scoring are labour-intensive, and prone to observer bias. This is especially true in modern livestock production systems, where physically identical animals are kept in increasingly large groups, making individual behavioural data even more challenging to obtain. As a consequence, our understanding of the genetics underlying welfare-related behavioural traits, particularly under (semi-)commercial conditions, remains limited. To address this issue, technologies like computer vision have been proposed to enable continuous behavioural phenotyping [10]. However, these systems bring their own challenges, including identity switches and false positive or negative detections [11] which could result in a systematic misassignment of phenotypes. While some studies have successfully estimated genetic parameters for behavioural traits in pigs using computer vision data [12], pigs are typically housed in smaller groups, are more visually distinguishable, and less prone to occlusion than poultry. This highlights the greater technical challenge that poultry poses for accurate individual tracking, especially in (semi-) commercial settings. Nevertheless, the advances in individual tracking now offer a critical opportunity to overcome one of the main barriers that has long impeded progress in the genetic improvement of animal welfare.

A potential solution to identity switches is the use of computer-readable markers, which are implemented in the publicly available software library called “ArUco” [13]. A study that used ArUco markers to identify and track for an extended period of time individual laying hens housed in groups of 10 individuals, yielded position data suitable for the measurement of activity and proximity of the individuals [14, 15].

In this research, we evaluated the suitability of ArUco-based tracking of individual laying hens for genetic analysis of behaviour-related traits, using birds housed under semi-commercial conditions. To our knowledge, this is the first study to perform genetic analysis of behaviour using individual-level ArUco tracking data in large groups of laying hens (> 100 hens) under semi-commercial housing conditions. We estimated the genetic parameters of three data-derived behavioural traits measured in the litter area. For each hour, the following three traits were obtained for each bird: detected or not, minutes detected and average walking speed. These traits were tested as potential proxies for activity in the litter area, a location which has been associated with behaviours that can indicate either positive or negative aspects of welfare. The estimation of significant genetic variance and of breeding values would demonstrate the existence of genetic effects on traits related to litter area activity, which could be potentially included in a breeding program.

## Methods

### Raw data

Hendrix Genetics (HG) provided a population of 1441 Dekalb White hens, all of the same age, and hatched at the same hatchery. The individuals were three-way crossbreds produced by artificially inseminating crossbred dams with pooled semen from pure-line sires. Genotypes from the custom-made Illumina 60 K SNP-chip for layers were also provided by HG.

**Fig. 1:**
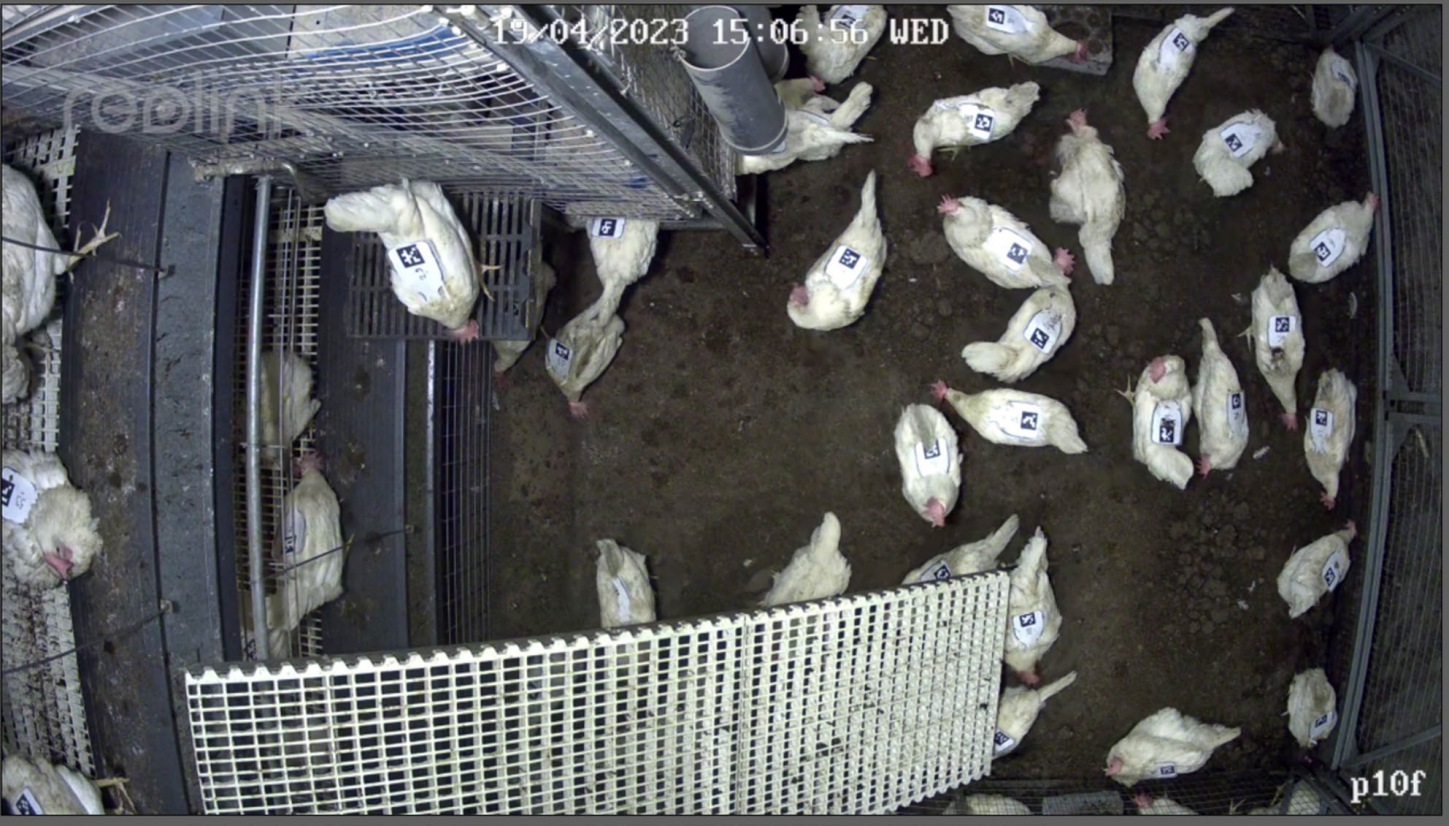
Example of camera’s view. On the top and on the bottom of the picture are the adjacent pens, to the right is the entrance to the pen, and on the left are the upper levels. The ramp going to upper levels can be seen on the bottom left

At 40 weeks of age, the individuals were housed in 12 pens located inside one barn, with approximately 120 hens per pen. Each pen measured 195 cm x 203 cm and consisted of three levels in an aviary design: the litter area on the ground level, the nesting area on the first level and the perching area on the second level. To reduce the chance of injuries due to jumps, a ramp going from the litter area to the perching area was integrated into the setup (Fig. 1).

**Fig. 2:**
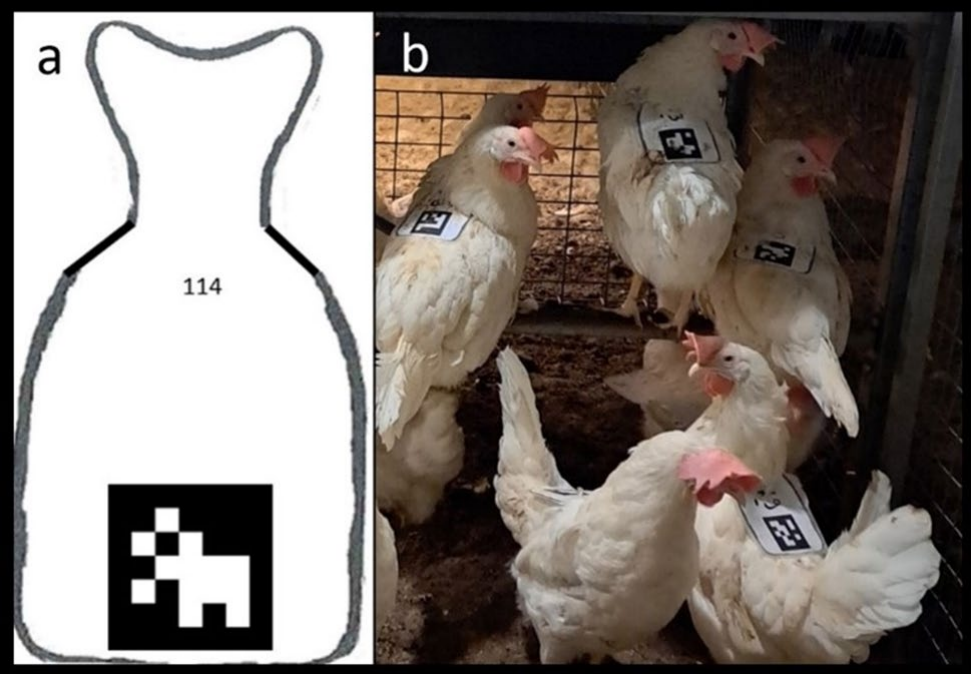
Example of backpacks and their placement. **a** Sheet of paper with backpack design and ArUco marker number 114; **b** Example of backpacks after placement

Within each pen, each individual was uniquely identified using an ArUco marker and a wing-tag. For this purpose, 150 different ArUco markers were generated using the 6 × 6 dictionary [16] selected as a practical compromise between marker uniqueness, detection robustness, and feasibility under semi-commercial conditions. Standard OpenCV ArUco dictionaries support up to 1000 distinct markers across grid sizes ranging from 4 × 4 to 7 × 7. Each marker was printed on a sheet of paper containing a template for a lightweight ergonomic backpack (Fig. 2a). The backpacks were then cut, laminated, and provided with elastic straps that worked as suspenders.

Sixteen weeks after housing, following the completion of a dietary trial conducted with the same population, the backpacks were placed on all individuals (Fig. 2b) for identification purposes during automated video tracking. Each individual’s wing-tag number and its corresponding ArUco number were recorded during the placement of the backpacks. ArUco markers were uniquely assigned within pen but were repeated across pens. The cameras were strategically positioned to minimize the possibility of detecting markers from adjacent pens (Fig. 1).

Each pen was equipped with one surveillance camera (B800, Reolink, Delaware, USA) positioned to overlook the litter area (Fig. 1). Hence, birds were recorded in the litter area only; the nesting and perching areas were not monitored. Consequently, birds were in the view of the camera only part of the time, and moved in and out of the camera’s field of view as they entered or left the litter area. The activity of the hens was recorded for 15 h in every pen over seven consecutive days. Due to technical disruptions at the farm, the total number of recorded hours varied from pen to pen, ranging from a total of 27 to 92 recorded hours per pen (mean = 55.7 h). Videos were recorded at a resolution of 2304 × 1296 pixels and 20 frames per second.

The coordinates of the four corners of every ArUco detected in each frame were obtained following the methodology in [14]. The resulting CSV file included the number of each detected ArUco marker, the x and y coordinates for each corner of each detected ArUco, the number of the video frame, the pen number, and the time and date. The individuals’ unique wing-tag numbers were incorporated into the file, using the backpack placement records. The position of the individuals in each detection frame was calculated as the average of the x and y coordinates of each of the corners of the marker. All editing and statistical analysis were performed using scripts written in the R 4.4.1 programming language [17].

### Filtering and final data

To enhance the reliability of the tracking data, a multi-step filtering protocol was applied within each pen.

**Fig. 3:**
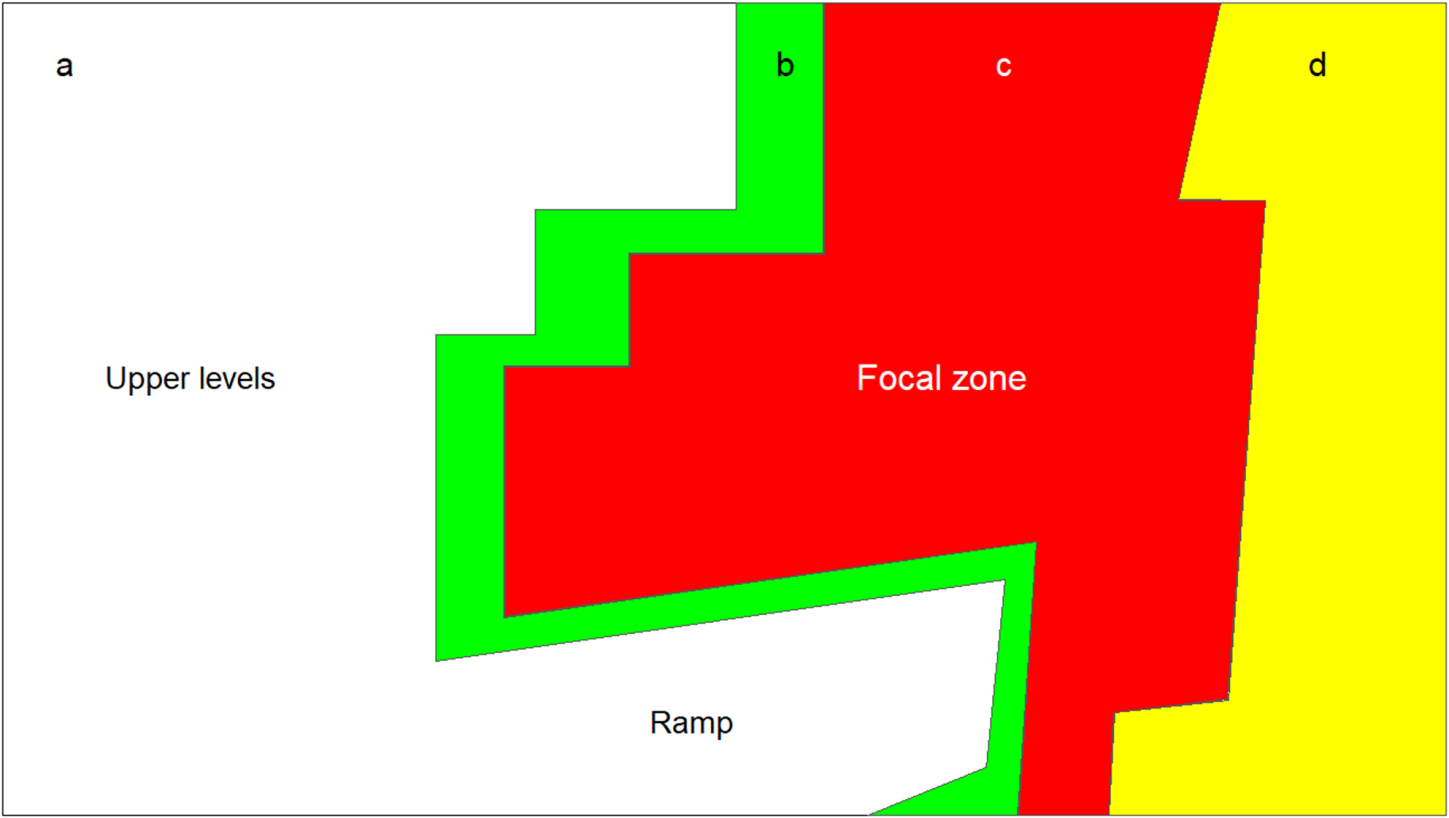
Diagram of zones for detection loss. **a** Exclusion zone: Area outside the monitoring range; **b** Green zone: Detection loss attributed to individuals leaving the detection zone; **c** Focal zone: Region where markers were expected to be clearly visible and detection failure were likely due to occlusion or limitations on detection power; **d** Yellow zone: Detection loss likely caused by excessive distance to the marker or lens distortion

First, since this study specifically examined activity within the litter area, any ArUco marker detected on the upper levels or on the ramp was excluded. To delimit the area of exclusion, the R package “sf” version 1.0–17 [18] was used to create a polygon that spatially bounded the possible detection coordinates of markers found on the upper levels and on the ramp (Fig. 3a).

Over time, wear and tear, such as dirt or bending of the backpackss, could alter the appearance of the markers, leading to the detection of ArUco markers that were not actually present in the pen. To mitigate this, only markers documented during the initial backpack placement were retained.

The first step of the algorithm in the detection of ArUco markers was the localization of the four corners of each marker. This methodology could lead to the detection of false positives. These could occur, for example, if the algorithm mistakenly detected corner-like patterns at opposite ends of the pen, which would be incorrectly interpreted as a single, and impossibly large marker. To address this, the physical area of each marker was calculated from its corner coordinates, and extreme outliers, determined via a visual inspection of a histogram (showing a right-skewed distribution, data not shown), were removed. Additionally, any marker appearing in multiple locations within the same millisecond was discarded. Moreover, to eliminate extreme movements that could result from tracking artifacts or identity swaps (i.e. two individuals being recognised as one), we applied a distance-based filter (see Traits for information on distance calculation). Distances moved within 10‑second windows were examined, and extreme outliers identified via visual inspection of a histogram were excluded (distribution skewed to the right, data not shown).

Next, videos from pens 1 and 9 were excluded because their recording resolution was 3840 × 2160 pixels instead of 2304 × 1296 pixels. Downscaling these videos to a common resolution would have been highly time‑consuming, and the data from the remaining pens was considered sufficient to continue with the analysis. Excluding the pens ensured consistent spatial scaling, avoiding artifacts that would arise from extracting marker coordinates from frames of different dimensions.

Table 1 shows a summary of the final available data for analysis after all filtering steps described above were performed.

**Table 1:** Description of available data after filtering

Total number of pens	10
Total number of recording days	7
Average number of recorded hours per pen-day	10.5
Total number of individuals	1132
Total number of genotypes	1132
Total number of detections	333,265,215
Average number of detections per individual per hour	5,326

Average number of recorded hours per pen-day: The mean number of hours recorded for each pen per day; Total number of individuals: Total number of detected individuals; Total number of genotypes: Total number of detected individuals that had genotypes; Total number of detections: The sum of all ArUco marker readings across all individuals and all video recordings; Average detections per individual per hour: The mean number of times each uniquely tagged hen was detected per hour

### Data quality test

Given the novelty of using ArUco marker-derived tracking data for genetic analysis, we first conducted a quality assessment to evaluate the dataset’s suitability for variance component estimation.

We assessed the tracking performance by measuring the total time individuals were effectively tracked, and evaluated the possible reasons for the non-detection periods. Non-detections were considered to occur primarily for three reasons: (1) individuals were outside that camera’s field of view (e.g. on the upper levels), (2) markers that were physically present and expected to be fully visible were not recognized by the system, or (3) the markers were positioned either too far from the camera or in areas that were affected by lens distortion (particularly at the image periphery, where wide-angle distortion occurs), which made their patterns unreadable. This distinction helped us differentiate between detection failures occurring under theoretically optimal conditions from those resulting from technical constraints or by individuals simply being outside the litter area.

The total minutes of detection was calculated for each individual, each hour of each day (individual-day-hour) as:

1 $$Minutes\;~detected = ~\left( {\frac{{Number\;~of\;frames\;where\;~detected}}{{20\;frames/{\mathrm{sec}}~*~60~sec/min}}} \right),$$

the remaining time in each individual-day-hour interval was classified as non-detection time. For example, if individual 13 was detected 45 min between 14:00 and 15:00 on the 18th of April, the non-detected time for that hour was 15 min.

Proportions of time detected and non-detected were calculated for every individual-day-hour. To obtain individual-level overall metrics, these hourly proportions were averaged within each individual over the 7-day recording period. Then, these individual averages were averaged across all individuals to obtain population-level estimates of time detected and non-detected.

To further understand the causes of non-detections, we specifically investigated instances when the tracking of an individual was interrupted, hereafter referred to as “detection-loss events” (DLEs). We wanted to determine whether these interruptions were linked to true absences of the individuals, to camera-related image distortion or to the system simply failing to detect markers that were expected to be clearly visible. For each DLE, we recorded the coordinates where the marker was last seen. This allowed us to examine whether these events were more likely to occur at the area where birds moved from the litter to the ramp or the upper levels, at the edges of the camera’s field of view (where distortion is strongest), or in the central area (where occlusion was more likely the cause of non-detection).

The DLEs were defined as instances where an individual, previously detected for at least one continuous minute, underwent a non-detection period of at least 70 consecutive frames (~ 3.5 s). The time span of a DLE was calculated as the quotient of the difference between the indexes of the video frame (i.e., the frame number) when it was last seen and the video frame when it was re-detected, over the frame rate of 20. For example, if an individual was last detected in frame number five and then re-detected in frame number 75, the length of that DLE would be $\frac{75-5}{20}=3.5sec$.

To analyse the spatial patterns in the loss of tracking, we divided the litter area into three distinct zones using the R package “sf” (Fig. 3b, c and d). This zoning approach was designed to differentiate the probable causes of detection failures. It helped distinguish if failures were more likely due to true absences, limitations in the detection power of the tracking algorithm or due to suboptimal camera placement and coverage (which led to image distortion or markers being too far away to be read). The zones were defined as follows:

- Green Zone (Fig. 3b): In this area, detection loss was most likely caused by hens moving to locations outside the detection area, such as jumping to upper levels, walking under the system, or moving onto or beneath the ramp.
- Yellow Zone (Fig. 3d): Detection loss in this area was likely related to technical limitations of the camera, such as image distortion near the edges of the field of view or excessive distance to markers that reduced their clarity. In these cases, the marker was within view, but it was difficult for the algorithm to detect it accurately.
- Focal Zone (Fig. 3c): In this central area, individuals were directly beneath the camera, where conditions for detection were expected to be optimal (minimal distortion and good coverage). Here, detection loss was likely caused by short-term occlusions (e.g., other birds blocking the marker) or due to possible limitations in the detection power of the tracking algorithm.

For each DLE, we recorded both the disappearance and the reappearance zones. This allowed us to categorize the total non-detection time into three distinct categories based on their most probable cause:

- “Away” events: In these events, both disappearance and reappearance occurred outside the Focal zone (i.e. green and/or yellow zones; Fig. 3b and d). These events were most likely caused by individuals physically moving out of the detection zone (i.e., being “away” from the detectable area), rather than by technical detection errors.
- “Uncertain” events: In these events, either the disappearance or the reappearance occurred within the Focal zone, while the other occurred in the green or yellow zones. These events may have resulted from individuals being within the detection area but located in parts where image distortion or excessive distance from the camera hindered marker recognition. Hence, it remained “uncertain” whether the individual was within the detection zone during the DLE.
- “Focal” events: For these events, both disappearance and reappearance occurred within the Focal zone, where markers were expected to be clearly visible and undistorted. Therefore, detection loss in these events was likely caused by temporary occlusion of the markers or limitations in the detection algorithm itself.

For each bird, hourly proportions of time detected and non-detected were calculated. The non-detected proportion was then subdivided into the “Away,” “Uncertain,” and “Focal” categories. These hourly proportions were averaged across the 7 days to obtain individual-level averages. Furthermore, population-level estimates were calculated by averaging individual means across all birds. In addition, the hourly rate of disappearances and reappearances within the focal zone was quantified for each individual.

### Genetic analysis

#### Within and between pen relationships

All individuals were genotyped using a custom Illumina 60 K SNP chip co‑developed by HG and Cobb‑Vantress for layers. As no pedigree information was available, a genomic relationship matrix (GRM) was calculated. Before GRM construction, genotype quality control (QC) was performed using PLINK 1.9 [19]. We retained 44,813 SNPs located on autosomal chromosomes after applying filters for a minor allele frequency (MAF) > 0.01 and a genotyping rate > 0.90 per SNP. The GRM was calculated from the QC-pruned genotypes using VanRaden’s first method [20] as implemented in the calc_grm software [21].

To ensure that no unintended genetic substructures affected the estimation of genetic parameters and breeding values (EBVs), we first assessed whether individuals housed in the same pen were, by chance, more genetically related to each other than those housed in different pens. This is important because substructures within the population, such as groups of more closely related individuals being housed together, can confound genetic effects with environmental effects associated with those pens. If not properly accounted for, such stratification could lead to biased heritability estimates and EBVs, not only for group-level traits [22, 23], but also for individual-level traits [24, 25], though the inclusion of a pen effect in the model should prevent such bias for individual-level traits.

To evaluate this, we used the GRM to calculate the average within- and between-pen relationships. The diagonal elements of the GRM, representing the relationships of individuals with themselves, were removed from the calculations. The pairwise genomic relationships between individuals were then grouped according to the pens in which the individuals were housed, and the average genomic relationship for all possible pen combinations were examined (e.g., relationships within pen 2, and between individuals in pens 2 and 3, 2 and 4, 3 and 4, 3 and 5, etc.). This approach allowed us to verify whether the mean genomic relationship within pens or between specific pens was substantially higher than expected, which could indicate a potential source of bias in our analyses.

#### Traits

From the tracking data, we derived three phenotypic traits for genetic analysis: one binary trait and two continuous traits. Both continuous traits were square root transformed to better approximate a normal distribution:

- Detected_hour (01_DH): A binary trait indicating whether an individual was detected in the litter area for at least 1200 consecutive frames (1 min) during a given day-hour interval (1 = detected at least once; 0 = not detected).
- Minutes_detected_hour (MDH): A continuous trait representing the square root of the total time (in minutes) that an individual was detected in the litter area per day-hour calculated as described in Eq. (1).
- Walking_speed_hour (WSH): A continuous trait quantifying hourly movement of an individual. This trait was obtained by summing the Euclidean distances between individual positions across consecutive frames. The distances calculated during DLEs (between the coordinates of disappearance and reappearance frames) were excluded from the sum. Next, the obtained distance was transformed to cm (8 pixels/cm conversion factor). The total Euclidean distance was divided by the MDH of the corresponding hour to obtain the average walking speed, and finally square root transformed.

A fixed pixel to cm conversion ratio was used for simplicity. All cameras were mounted in the same top‑down position, reducing perspective variation across pens. Although lens distortion and perspective may cause local variation in this ratio, any systematic error affects all hens within the same camera view equally, thereby preserving rankings between individuals. Since our genetic analysis relies on phenotypic covariances between individuals, this simplification was not expected to bias the estimation of genetic parameters.

Whenever an individual had zero as record for 01_DH in a given day-hour, the observations for MDH and WSH were coded as a missing value (“NA”). Table 2 shows an example of the resulting data.

**Table 2:** Example of structure of data set with phenotypic observations before trait transformation

ArUco	Wingtag	Hour	Pen	Day	01_DH	MDH (min)	WSH (cm/min)
114	9661	10	2	18	1	8	250.12
112	8924	10	2	18	0	NA	NA
99	6802	10	2	18	0	NA	NA
9	8608	10	2	18	1	7	194.9

ArUco: number from ArUco library; Wingtag: unique individual identity number; Hour: time of the day; Pen: number of pen; Day: date; 01_DH: detected or not; MDH: minutes detected per hour, WSH: walking speed per hour (cm/min)

#### Univariate analysis

The estimation of variance components, genetic parameters, and breeding values was performed using a genomic REML in ASReml 4.2.1 software [26], which employs the Average Information algorithm for REML estimation.

Each trait was analysed separately using a univariate LMM. Fixed effects included pen, day and the hour of the day. Random effects included pen-day-hour interaction, the permanent environmental effect of the animal, and the genetic effect of the animal. Pen, day, and hour were fitted as fixed effects due to their low number of levels (Table 1) and high number of observations per level, enabling precise correction for systematic differences without overfitting. Their interaction was included as a random effect because it comprises many levels with fewer observations per level. This approach shrinks the interaction effects toward zero and treats them as structured residual noise, thereby preventing overfitting. Fitting main effects as fixed thus reduces environmental variance, enhancing precision of random genetic estimates while the interaction effect prevents overfitting.

The model can be represented as:

2 $${\mathbf{y}} = {\mathbf{Xb}} + {\mathbf{Za}} + {\mathbf{Vc}} + {\mathbf{Wd}} + {\mathbf{e}}$$

where **y** is the vector of observations of the trait under analysis; **X** is the incidence matrix connecting records to fixed effects; **b** is the vector of fixed effects: pen, day, and hour; **Z** the incidence matrix connecting each individual’s phenotype to its own breeding value; **a** is the vector of breeding values; **V** is the incidence matrix connecting records to the random pen-day-hour interaction effect; **c** is the vector of independent pen-day-hour effects; **W** is the incidence matrix connecting records to the permanent random effect of the animal; **d** is the vector with permanent random effects of the animal; and **e** is the vector of residuals. The distribution of the random effects is $\mathbf{a}\sim\mathrm{N}(0,\mathbf{G}{{\upsigma}}_{\mathrm{A}}^{2})$, $\mathbf{c}\sim\mathrm{N}(0,\mathbf{I}{{\upsigma}}_{\mathrm{c}}^{2})$, $\mathbf{d}\sim\mathrm{N}(0,\mathbf{I}{{\upsigma}}_{\mathrm{d}}^{2})$, where **G** is the GRM, and $\mathbf{I}$ an identity matrix. To consider the natural differences of the individuals‘ activities during the day, a separate residual variance was estimated for each pen-hour group using ${\mathbf{e}}_{\mathbf{k}}\sim\mathrm{N}(0,\mathbf{I}{{\upsigma}}_{{\mathrm{e}}_{\mathrm{k}}}^{2})$, where k was the index of the pen-hour group. Heteroskedasticity across days was also assessed, but was considered to be too small to model (results not shown).

#### Heritability and coefficient of genetic variation

The inclusion of the pen–day–hour variance in the phenotypic variance denominator reflects our decision to define total phenotypic variance as the sum of all modelled random components, rather than only genetic and residual variances. While some heritability formulations exclude systematic environmental effects from the phenotypic variance, here the pen–day–hour interaction represents a source of environmental variation that would otherwise be absorbed into the residual if not modelled explicitly. The estimable variance of this effect depends on the scale of measurement (e.g., it differs between hourly and daily records), and any unmodeled portion would be allocated to the residual variance, which invariably contributes to the heritability denominator. Therefore, we estimated heritability as the proportion of variance explained by additive genetic effects relative to the total phenotypic variance of the traits, which includes the pen–day–hour interaction.

The heritability of the hourly traits was calculated:

3 $${\mathrm{h}}_{{{\mathrm{hour}}}}^{2} = \frac{{\sigma _{{\mathrm{a}}}^{2} }}{{\left( {\sigma _{{\mathrm{a}}}^{2} + \sigma _{{\mathrm{c}}}^{2} + \sigma _{{\mathrm{d}}}^{2} + \bar{\sigma }_{{\mathrm{e}}}^{2} } \right)}},$$

where $\stackrel{-}{{{\upsigma}}_{\mathrm{e}}^{2}}$ denotes the residual variances averaged over all pen-hour groups.

Heritability estimates for each trait were also calculated on a daily basis, i.e. referring to the individuals’ mean performances averaged across all recorded hours per day. To obtain these estimates, the variance components previously estimated from the hourly records were adjusted to reflect the averaging of repeated measurements. Because the genetic and permanent environmental effects of an individual are the same across the hours of the day, their variance remains unchanged when considering the daily mean performance. In contrast, the pen-day-hour and residual effects are specific to each hourly observation and, therefore, their variances were divided by the average number of recorded hours per pen-day (10.5 h; see Table 1). This adjustment reflects the reduction in variance that occurs when averaging repeated measures:

4 $${\mathrm{h}}_{{{\mathrm{day}}}}^{2} = \frac{{\sigma _{{\mathrm{a}}}^{2} }}{{\left( {\sigma _{{\mathrm{a}}}^{2} + \frac{{\sigma _{c}^{2} }}{{10.5}} + \sigma _{{\mathrm{d}}}^{2} + \frac{{\bar{\sigma }_{{\mathrm{e}}}^{2} }}{{10.5}}} \right)}}$$

where ${\mathrm{h}}_{\mathrm{d}\mathrm{a}\mathrm{y}}^{2}$ is the heritability of mean performances.

The coefficient of genetic variation (GCV) of each trait was calculated as the additive standard deviation of the trait divided by the population (phenotypic) mean of the same trait.

#### Reported accuracy of estimated breeding values

Average reported accuracies for each trait were calculated using the standard errors of the individual EBVs obtained from the univariate LMMs, as reported by ASReml in the .sln output file.

5 $$\rho _{{\hat{a}}} = {\mathrm{average}}\left( {\sqrt {\frac{{\widehat{{\sigma _{a}^{2} }} - ~SE_{i}^{2} }}{{\widehat{{\sigma _{a}^{2} }}}}} } \right),~$$

where ${{\uprho}}_{\widehat{\mathrm{a}}}$ is the accuracy of EBV averaged over individuals, $\widehat{{{\upsigma}}_{\mathrm{a}}^{2}}$ the estimated additive genetic variance of the trait, and ${\mathrm{S}\mathrm{E}}_{\mathrm{i}}$ the standard error of the EBV of individual i as reported by ASReml.

#### Trivariate analysis

A trivariate analysis was performed to estimate the phenotypic and genetic correlations between traits, and to investigate whether accounting for censoring on 01_DH would improve EBVs accuracy. This censoring is inherent to the data structure (Table 2), as the other two traits were only observed for individuals with a value of 01_DH equal to 1.

The model for the trivariate analysis was similar to the univariate models Eq. (2), but due to the model not converging, the heterogeneous residual variance was removed. The model can be expressed as:

6 $$\begin{aligned} \left[ {\begin{array}{*{20}c} {\begin{array}{*{20}c} {{\mathbf{y}}_{1} } \\ {{\mathbf{y}}_{2} } \\ \end{array} } \\ {{\mathbf{y}}_{3} } \\ \end{array} } \right] = & \left[ {\begin{array}{*{20}c} {{\mathbf{X}}_{1} } & 0 & 0 \\ 0 & {{\mathbf{X}}_{2} } & 0 \\ 0 & 0 & {{\mathbf{X}}_{3} } \\ \end{array} } \right]\left[ {\begin{array}{*{20}c} {\begin{array}{*{20}c} {{\mathbf{b}}_{1} } \\ {{\mathbf{b}}_{2} } \\ \end{array} } \\ {{\mathbf{b}}_{3} } \\ \end{array} } \right] \\ & + \left[ {\begin{array}{*{20}c} {{\mathbf{Z}}_{1} } & 0 & 0 \\ 0 & {{\mathbf{Z}}_{2} } & 0 \\ 0 & 0 & {{\mathbf{Z}}_{3} } \\ \end{array} } \right]\left[ {\begin{array}{*{20}c} {\begin{array}{*{20}c} {{\mathbf{a}}_{1} } \\ {{\mathbf{a}}_{2} } \\ \end{array} } \\ {{\mathbf{a}}_{3} } \\ \end{array} } \right] + \left[ {\begin{array}{*{20}c} {{\mathbf{V}}_{1} } & 0 & 0 \\ 0 & {{\mathbf{V}}_{2} } & 0 \\ 0 & 0 & {{\mathbf{V}}_{3} } \\ \end{array} } \right]\left[ {\begin{array}{*{20}c} {\begin{array}{*{20}c} {{\mathbf{c}}_{1} } \\ {{\mathbf{c}}_{2} } \\ \end{array} } \\ {{\mathbf{c}}_{3} } \\ \end{array} } \right] \\ & + \left[ {\begin{array}{*{20}c} {{\mathbf{W}}_{1} } & 0 & a \\ 0 & {{\mathbf{W}}_{2} } & 0 \\ 0 & 0 & {{\mathbf{W}}_{3} } \\ \end{array} } \right]\left[ {\begin{array}{*{20}c} {\begin{array}{*{20}c} {{\mathbf{d}}_{1} } \\ {{\mathbf{d}}_{2} } \\ \end{array} } \\ {{\mathbf{d}}_{3} } \\ \end{array} } \right] + \left[ {\begin{array}{*{20}c} {\begin{array}{*{20}c} {{\mathbf{e}}_{1} } \\ {{\mathbf{e}}_{2} } \\ \end{array} } \\ {{\mathbf{e}}_{3} } \\ \end{array} } \right] \\ \end{aligned}$$

where ${\mathbf{y}}_{1}$, ${\mathbf{y}}_{2}$, and ${\mathbf{y}}_{3}$ represent a vector of observations of traits 01_DH, MDH, and WSH, respectively, and the rest of the terms are analogous to the univariate LMM.

The distribution of the genetic effects was:

7 $$\left[ {\begin{array}{*{20}c} {\begin{array}{*{20}c} {{\mathbf{a}}_{1} } \\ {{\mathbf{a}}_{2} } \\ \end{array} } \\ {{\mathbf{a}}_{3} } \\ \end{array} } \right] = N\left( {\left( {\begin{array}{*{20}c} {\begin{array}{*{20}c} 0 \\ 0 \\ \end{array} } \\ 0 \\ \end{array} } \right),\left[ {\begin{array}{*{20}c} {\sigma _{{{\mathrm{a}}_{1} }}^{2} } & {\sigma _{{{\mathrm{a}}_{1} {\mathrm{a}}_{2} }} } & {\sigma _{{{\mathrm{a}}_{1} {\mathrm{a}}_{3} }} } \\ {\sigma _{{{\mathrm{a}}_{1} {\mathrm{a}}_{2} }} } & {\sigma _{{{\mathrm{a}}_{2} }}^{2} } & {\sigma _{{{\mathrm{a}}_{2} {\mathrm{a}}_{3} }} } \\ {\sigma _{{{\mathrm{a}}_{1} {\mathrm{a}}_{3} }} } & {\sigma _{{{\mathrm{a}}_{2} {\mathrm{a}}_{3} }} } & {\sigma _{{{\mathrm{a}}_{3} }}^{2} } \\ \end{array} } \right] \otimes {\mathbf{G}}} \right),$$

where ${{\upsigma}}_{{\mathrm{a}}_{\mathrm{i}}}^{2}$is the genetic variance of ${\mathbf{y}}_{\mathrm{i}}$ and ${{\upsigma}}_{{\mathrm{a}}_{\mathrm{i}}{\mathrm{a}}_{\mathrm{j}}}$ the genetic covariance between ${\mathbf{y}}_{\mathrm{i}}$ and ${\mathbf{y}}_{\mathrm{j}}$; for i, j = 1,2 and 3. **G** is the GRM as used for the univariate analyses described above.

The distribution of the pen-day-hour effect was:

8 $$\left[ {\begin{array}{*{20}c} {\begin{array}{*{20}c} {{\mathbf{c}}_{1} } \\ {{\mathbf{c}}_{2} } \\ \end{array} } \\ {{\mathbf{c}}_{3} } \\ \end{array} } \right] = N\left( {\left( {\begin{array}{*{20}c} {\begin{array}{*{20}c} 0 \\ 0 \\ \end{array} } \\ 0 \\ \end{array} } \right),\left[ {\begin{array}{*{20}c} {\sigma _{{{\mathrm{c}}_{1} }}^{2} } & {\sigma _{{{\mathrm{c}}_{1} {\mathrm{c}}_{2} }} } & {\sigma _{{{\mathrm{c}}_{1} {\mathrm{c}}_{3} }} } \\ {\sigma _{{{\mathrm{c}}_{1} {\mathrm{c}}_{2} }} } & {\sigma _{{{\mathrm{c}}_{2} }}^{2} } & {\sigma _{{{\mathrm{c}}_{2} {\mathrm{c}}_{3} }} } \\ {\sigma _{{{\mathrm{c}}_{1} {\mathrm{c}}_{3} }} } & {\sigma _{{{\mathrm{c}}_{2} {\mathrm{c}}_{3} }} } & {\sigma _{{{\mathrm{c}}_{3} }}^{2} } \\ \end{array} } \right] \otimes {\mathbf{I}}} \right),$$

the distribution of the permanent random effect of the animal, and the residuals were analogous to those of the pen-day-hour effect.

After fitting the initial model, small residual covariances between 01_DH and the other traits were observed: WSH showed a covariance of 0.01 with 01_DH, and MDH showed a covariance of -0.02 with 01_DH (data not shown). These covariances were considered artefactual, because observations for MDH and WSH were missing for non-detected animals (i.e., for those with 01_DH equal to zero). For this reason, all animals with non-missing records on MDH and WSH had a value of one for 01_DH, resulting in no information in the data to estimate residual correlations with 01_DH. The observed estimates likely arose from the estimation of the fixed effects for 01_DH. Therefore, it was decided to re-run the model with the following constraints: the previously estimated residual variances for each trait were fixed, the residual covariance between WSH and MDH was also fixed to its estimated value, and the residual covariances involving 01_DH were fixed to zero. All other variance components were re-estimated and subsequently used for calculating heritabilities, accuracies, and EBVs.

#### Cross-validation accuracy of estimated breeding values

For cross-validation, the variance components used were those estimated with the LMMs in ASReml, and the EBVs were obtained using MiXBLUP software [27]. Cross-validation was performed for EBVs from both univariate and trivariate models.

A 10-fold cross-validation was performed by iteratively masking phenotypic data from one unique pen, while predicting EBVs for individuals in that pen (“EBVs from Masked Phenotypes”, EBVMP) using the phenotypes from the nine remaining pens.

The models used for the cross-validation were analogous to those used during the estimation of variance components (Eqs. (2) and (6)), but with fixed variance parameters (including all $\widehat{{\sigma}_{e}^{2}}$), to maintain consistency with the training set EBVs. Reliabilities for EBVMP reported by MiXBLUP were square root transformed to obtain reported accuracies, which were later compared to the accuracy estimated with the cross-validation.

The methodology used to estimate accuracy with cross-validation was as follows:

To evaluate the dispersion and accuracy of resulting EBVs while accounting for other systematic and random effects, we fitted LMMs in ASReml, including a fixed regression of records on the EBVMP. In these models, the random genetic effect of the animal was replaced by the EBVMP covariate, and variance components were re-estimated.

9 $$\begin{aligned} y_{{ijkl}} = & Pen_{i} + Hour_{j} + Day_{k} \\ & + EBVMP_{l} *\beta + PDH_{{ijk}} + PE_{l} + e_{{ijkl}} \\ \end{aligned}$$

where EBVMP is a fixed covariate for the animal’s EBV of masked phenotypes with slope $\beta$, PDH is the random effect of the interaction between the pen *i*, the hour *j*, and the day *k*, and PE is the random permanent effect of individual *l*. The dispersion of EBVMP was measured by the estimate of β, where $\widehat{\beta}$ =1 indicates correct dispersion, $\widehat{\beta}$ < 1 overdispersion of EBV, and $\widehat{\beta}$> 1 underdispersion of EBV (e.g. [28, 29]).

The accuracy of EBVMP $\left({\rho}_{EBVMP}\right)$ was estimated using the relationship.

10 $$\rho _{{EBVMP}} = \frac{{\rho \left( {EBVMP,\;\tilde{y}} \right)}}{h},$$

where ρ(EBVMP, $\stackrel{\sim}{y}$) is the correlation between the EBVMPs and the phenotypic observations corrected by fixed effects ($\stackrel{\sim}{y}$), and h is the square root of the trait’s heritability. The $\rho(EBVMP,\stackrel{\sim}{y})$ was calculated as the square root of the proportion of variance in $\stackrel{\sim}{y}$ explained by the regression on EBVMP. Thus, the accuracy of EBVMP was calculated as.

11 $$\rho _{{EBVMP}} = \frac{{\sqrt {\frac{{\hat{\beta }^{2} \sigma _{{EBVMP}}^{2} }}{{\hat{\beta }^{2} \sigma _{{EBVMP}}^{2} + \widehat{{\sigma _{c}^{2} }} + \widehat{{\sigma _{d}^{2} }} + \overline{{\widehat{{\sigma _{e}^{2} }}}} }}} }}{h},$$

where the numerator represents the square root of the proportion of variance in $\stackrel{\sim}{y}$ explained by EBVMP, $\widehat{\beta}$ the estimated fixed regression coefficient of the phenotypes on EBVMP from the validation model, ${\sigma}_{EBVMP}^{2}$ the variance of the EBVs of masked phenotypes, hats denote estimates, and $\stackrel{-}{\widehat{{\sigma}_{e}^{2}}}$ the residual variance averaged over pen-hour estimates.

For the trivariate model, the regression on EBVMP was first run with all variance components freely estimated. Subsequently, residual variances for each trait and the residual covariance between WSH and MDH were fixed at their estimated values. Residual covariances involving 01_DH were fixed to zero, as explained in the trivariate analysis section above. This fixed-variance model was used to obtain the variance components necessary for the calculation of cross-validation accuracy.

## Results

### Data quality test

For each individual, the number of detection losses in the focal zone was calculated for each detected hour and then averaged across all detected hours. This individual-level average varied considerably among birds, ranging from 1.0 to 135.27 (Additional File 1 Fig. S1; median = 19.3, mean = 23.4, SD = 17.8). Additional File 1 Fig. S1 shows a right-skewed distribution, indicating that detection loss occurred across most individuals rather than being concentrated in a small subgroup. While some hens experienced higher loss rates, the majority clustered around the mean and median values.

**Fig. 4:**
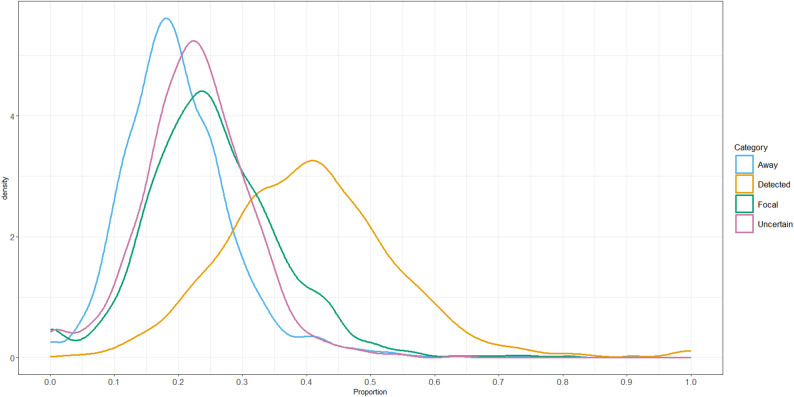
Density distribution of individuals’ average proportion of detected time and of DLE categories. Proportions were first calculated per hour, then averaged per day, and finally averaged across the seven days of video recordings. The density curves represent the estimated distribution of these individual-level averages

Figure 4 shows the density distribution of the average proportion of time that each individual spent in each DLE category over the 7-day recording period. Table 3 presents the corresponding population-level means.

On average, individuals were successfully detected 37% of the total observation time, with the proportion of time detected varying substantially among individuals. The “detected” category had a wide spread distribution, peaking around 0.45 and ranging from 0.2 to 0.8, with a small peak at 1.0. These results show that the majority of individuals were not continuously detected throughout the recordings, and that they experienced periods of non-detection.

The non-detection time, which includes the “Away”, “Uncertain”, and “Focal” categories, accounted for the remaining 63% of the total observation period. These categories varied in size and consistency. The Focal category was the largest, accounting for 28% of the non-detection time, with values that varied widely across individuals and peaked at approximately 0.25. By contrast, the Away category represented 15% of the total non-detection time and showed a sharp peak around its mean value, suggesting that missed detections due to birds being physically outside the camera’s detection zone were relatively uncommon and exhibited less variation across individuals than the other categories.

The Uncertain category represented 20% of the total non-detection time, with its density peaking around 0.22. This category likely captured a mixture of detection failures related to image distortion, distance from the camera, and partial occlusion at the edges of the field of view. Overall, these results indicate that most non-detection time occurred within the Focal and Uncertain zones, where birds were expected to be visible, rather than due to true absences from the monitored area.

**Table 3:** Population-level average proportions of detection and of non-detection categories

Detection and non-detection categories	Proportion
Detected	0.37
Away	0.15
Uncertain	0.20
Focal	0.28

Detected: Proportion of time individuals were successfully tracked; Away: Proportion of time indicating individuals likely left the litter area; Uncertain: Proportion indicating that individuals were likely in a sub-optimal area for detection (e.g. distorted or too far away); Focal: Proportion of indicating detection failure due to occlusion or limits in detection power

### Genetic analysis

**Fig. 5:**
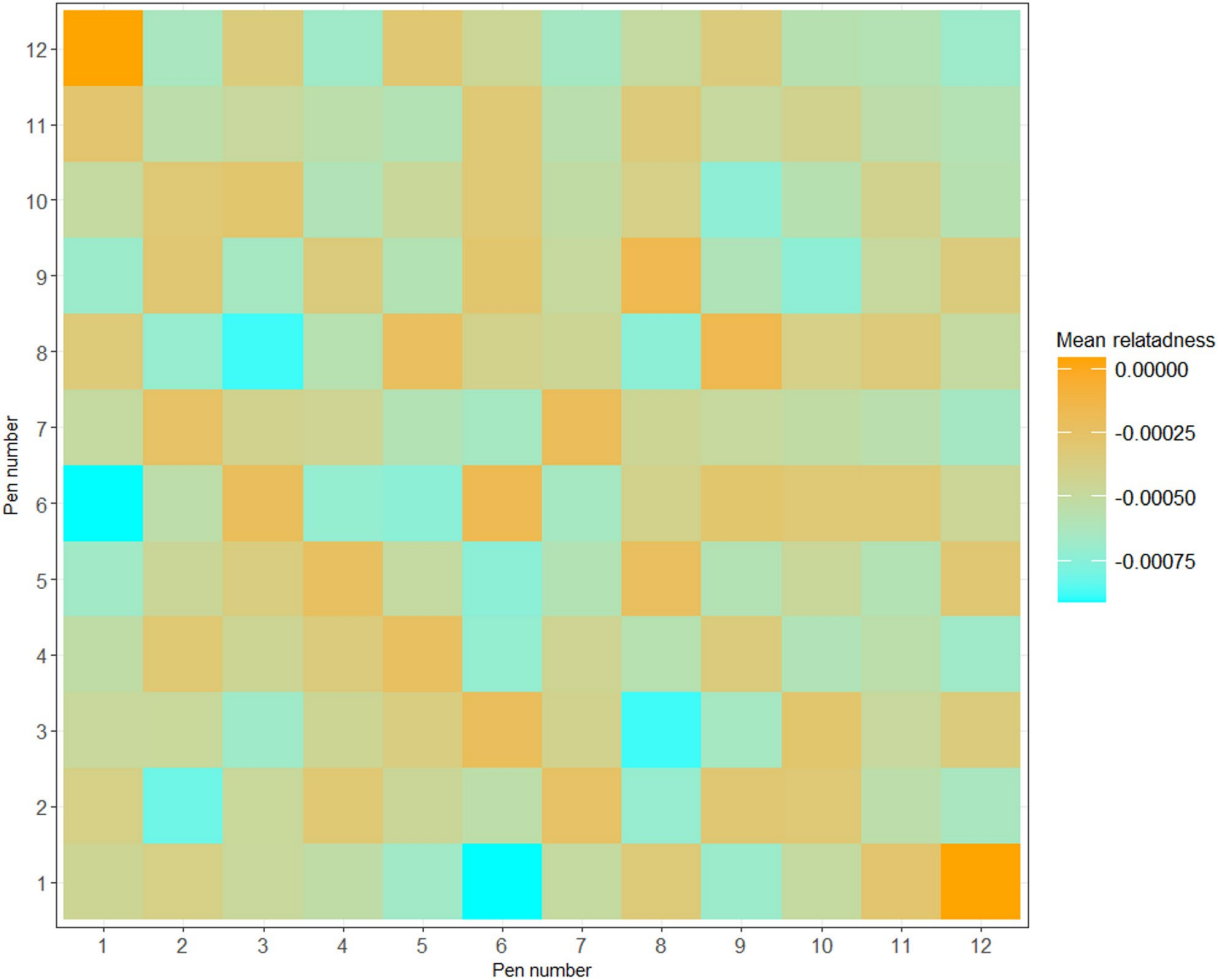
Mean relatedness within and between pens. Relatedness of individuals with themselves was not included in the calculation of the within pens mean relatedness

The genomic relatedness matrix across pens (Fig. 5) showed uniformly low values (–0.00075 to 0.00000), indicating minimal population stratification. The even distribution within and across pens confirmed that genetic backgrounds were well randomized, reducing concerns of confounding between pen and genetic effects. Importantly, the lack of elevated within-pen relatedness ensures that estimates of pen effects in our mixed models were not biased by clustering of related individuals.

Table 4 presents the phenotypic means, variance component estimates from univariate LMMs, and likelihood ratio test (LRT) results for genetic variance across traits. Table 5 shows heritability estimates based on hourly and daily measurements, the coefficient of genetic variation (GCV), and the average reported EBV accuracy across traits. All variance components and heritability estimates were significantly different from zero, and LRT results confirmed significant genetic variance for all traits. Heritability estimates were low when based on hourly measurements but increased approximately threefold when using daily measurements. Non genetic permanent individual variances were clearly larger than genetic variances, and repeatability was moderate for all traits. The GCV estimates were modest across traits, while reported EBV accuracies were moderately high.

Variance components estimated from the trivariate model (Additional File 2 Table S1) closely aligned with those from the univariate models (Table 4) for all traits. Heritability estimates from the trivariate model (Table 6, diagonal) also agreed with those from the univariate analysis (Table 5), suggesting consistency across modelling approaches. The phenotypic correlation was only calculated between MDH and WSH, which showed a strong negative value. Phenotypic correlations involving 01_DH were excluded because MDH and WSH were not observed (NA) when 01_DH equaled 0. Genetically, MDH exhibited significant correlations with both 01_DH and WSH. The strong negative correlations between MDH and WSH were unexpected, as these traits were hypothesized to reflect aspects of activity in the litter area (See Discussion).

Table 7 presents the results of the cross-validation using univariate and trivariate models. For all traits, both models produced very similar estimates for the regression slopes, and accuracies. Across all traits, the regression slopes on EBVMP were significantly different from zero, and not significantly different from one. This indicates that the EBVs adequately captured genetic variation without evidence of systematic over- or under-dispersion. Cross-validation accuracies were modest for all traits. In contrast, reported accuracies of EBVMP were approximately two times larger, suggesting over-estimation of the model-based accuracy. Notably, the trivariate model did not lead to a meaningful improvement in accuracy, despite accounting for genetic correlations between traits and selection on 01_DH. This suggests that, within this dataset, joint modelling provided no substantial advantage for improving EBV prediction compared to analyzing traits separately.

**Table 4:** Variance components estimates and phenotypic means for all traits

Traits	Mean	${\sigma}_{a}^{2}$ (SE)	LRT (*p*_value)	${\sigma}_{c}^{2}$ (SE)	${\sigma}_{d}^{2}$ (SE)	$\stackrel{-}{{\sigma}_{e}^{2}}$ (SE)	${\sigma}_{P}^{2}$
01_DH	0.60	0.01 (± 0.005)	7.6 (*p* < 0.01)	0.01 (± 0.0005)	0.05 (± 0.004)	0.16 (± 0.001)	0.23
MDH	2.7	0.11 (± 0.03)	18.2 (*p* < 0.01)	0.06 (± 0.005)	0.24 (± 0.02)	0.85 (± 0.07)	1.27
WSH	16.17	0.67 (± 0.24)	9.2 (*p* < 0.01)	0.51 (± 0.04)	2.26 (± 0.18)	8.56 (± 0.08)	12.00

Mean: population mean of the trait; 01_DH: detected or not per hour; MDH minutes detected per hour; WSH: walking speed per hour (in cm); ${\sigma}_{a}^{2}$ additive variance; LRT: likelihood ratio test for genetic variance, ${\sigma}_{c}^{2}$ variance of pen-day-hour effect; ${\sigma}_{d}^{2}$ variance of permanent animal effect; $\overline{\sigma }_{e}^{2}$: averaged residual variance; ${\sigma}_{P}^{2}$: phenotypic variance

**Table 5:** Heritability for hourly and daily measurements, GCV, repeatability, and average whole-data EBV reported accuracy

Traits	${h}_{hour}^{2}$ (SE)	${h}_{day}^{2}$ (SE)	Repeatability (SE)	GCV	Reported accuracy
01_DH	0.05 (± 0.02)	0.14 (± 0.05)	0.28 (± 0.01)	0.18	0.66
MDH	0.08 (± 0.02)	0.24 (± 0.06)	0.28 (± 0.01)	0.12	0.71
WSH	0.06 (± 0.02)	0.18 (± 0.06)	0.24 (± 0.01)	0.05	0.69

01_DH: detected or not per hour; MDH minutes detected per hour; WSH: walking speed per hour (in cm); GCV: coefficient of additive genetic variance; SE: standard error; Repeatability: (${\sigma}_{a}^{2}+{\sigma}_{d}^{2})/{\sigma}_{P}^{2}$(Table 4);${h}_{day}^{2}$: heritability using daily measurements; Reported accuracy: model-based accuracy.

**Table 6:** Genetic and phenotypic correlations between traits (SE), and heritability estimate for hourly measurements from the trivariate model

	01_DH	MDH	WSH
01_DH	0.05 (± 0.02)	–	–
MDH	0.73 (± 0.13)	0.08 (± 0.02)	−0.56 (± 0.01)
WSH	−0.28 (± 0.24)	−0.72 (± 0.13)	0.06 (± 0.02)

01_DH: detected or not per hour; MDH minutes detected per hour; WSH: walking speed per hour (in cm); Diagonal: heritability estimated with trivariate model; Lower off-diagonal: genetic correlations between traits; Upper off-diagonal: phenotypic correlations; SE: standard error

**Table 7:** Results of cross-validation

Trait	Univariate models			Trivariate model		
	$\widehat{\beta}$ (SE)	Validation accuracy	Reported accuracy	$\widehat{\beta}$ (SE)	Validation accuracy	Reported accuracy
01_DH	1.000 (0.278)	0.256	0.556	0.950 (0.225)	0.259	0.563
MDH	0.933 (0.178)	0.296	0.586	0.976 (0.172)	0.281	0.575
WSH	1.189 (0.246)	0.303	0.565	1.124 (0.212)	0.315	0.570

01_DH: detected or not per hour; MDH minutes detected per hour; WSH: walking speed per hour (in cm); EBVMP: Estimated breeding values for masked phenotypes; $\widehat{\beta}$: Regression slope of phenotypes on masked EBVMPs; Reported accuracy: average reported accuracy of EBVMPs

## Discussion

To our knowledge, this is the first study to successfully perform genetic analysis of behavioural traits derived from automated video tracking in a high-density, semi-commercial laying hen environment. Previous studies using ArUco markers have typically been conducted in smaller groups (< 20 birds) under controlled experimental conditions [14, 15]. While these studies demonstrated the feasibility of marker-based tracking for individual identification, they did not attempt genetic parameter estimation. In contrast, our study involved over 1,100 birds housed in groups of approximately 120 per pen, presenting significant challenges related to occlusion, marker visibility, and data loss. Despite these challenges, we were able to derive genetically informative phenotypes for detection and movement traits, and to demonstrate statistically significant genetic variation in behavioural traits.

The tracking data quality test revealed that individuals were detected on average 37% of the time. Genetic analyses revealed minimal population stratification and showed significant genetic variance across traits. Heritability was low when based on hourly measurements but increased substantially when considering the traits as daily measurements, and repeatability values were moderate across traits. The genetic correlation between MDH and WSH was strongly negative, which suggests that time spent in the litter and walking speed are genetically similar but expressed in opposite directions. Cross-validation showed that EBVs captured genetic variation without systematic over- or under-dispersion, however, validation accuracies were modest and lower than model-based values.

### Data quality test

Detection patterns in this study reveal a combination of both natural movement of birds and technical limitations in marker recognition. Notably, approximately half of the non-detection time occurred within the Focal zone (Table 3), where markers were expected to be visible. This suggests that many detection losses were not due to birds leaving the area but to challenges in consistently detecting markers under otherwise favourable conditions. Similar challenges have been reported in previous studies using computer-readable markers, where temporary obstructions (e.g., feathers, dustbathing behaviours) impair marker readability despite birds remaining within view [14]. Other reported causes for reduced marker readability are stains (e.g. dirt, manure), use of non-matte plastic during lamination, and sub-optimal camera placement that results in extreme angles or distances to the markers [15]. Future improvements, compared to the setup in the current study, could include printing larger markers or incorporating multiple markers per backpack to reduce occlusion-related detection loss, alongside more durable lamination materials and optimized camera placement to mitigate the technical limitations observed here.

Since non-detection frequently occurred even in the Focal zone, the interpretation of such tracking data should be cautious, as these periods may reflect technical rather than behavioural factors. However, despite these limitations, we achieved an average of over 5,000 detections per individual per hour (Table 1), which would be unattainable with manual annotation. Additionally, the distribution of hourly detection-loss rates in the Focal zone (Additional File 1 Fig. S1) indicates that detection interruptions were widespread across individuals, with most hens experiencing similar loss rates rather than the issue being confined to a small subset of birds. Future studies could also examine the duration of detection‑loss events by cause (e.g., Away, Uncertain, Focal), which may help distinguish between brief technical occlusions (e.g., feathers temporarily obstructing the marker) and longer, behaviour‑related absences from the litter area (e.g., movement to other levels).

In quantitative genetics, random noise in phenotypic observations is common and routinely accounted for through statistical models that partition observed variation into genetic and non-genetic components [30]. These models can produce reliable EBVs even when phenotypes are affected by environmental noise or measurement error, provided these errors are random and not systematically biased among individuals [31]. In this study, we treated detection losses as a source of residual variation, assuming they are largely independent of genotype. If this assumption holds, detection errors inflate residual variance without biasing genetic parameter estimates, preserving the utility of EBVs for comparative purposes [32]. However, if individual propensity for tracking loss were itself heritable, this would not be a mere artifact but part of the genetic architecture of what the MDH trait captures. In that case, the heritability estimate would refer to a composite trait that includes both the time spent in the litter area and the propensity of being detected. We cannot exclude this possibility, but we assume that such effects, if present, are small enough that EBVs for litter-area activity remain informative.

### Genetic analysis

This study shows that genomic analyses of behavioural traits derived from automated tracking data are feasible, though the accuracy of genomic predictions remains modest. The within- and between-pen genomic relatedness was not systematically different (Fig. 5), which indicates that individuals were distributed in a way that successfully avoided population stratification, and thereby reducing the risk of confounding pen and genetic effects [33]. The slightly negative mean within‑pen relationships (Fig. 5) arise because we excluded the diagonal from the calculation, and the genomic relationship matrix is relative to a mean of zero because allele frequencies were estimated from the sample [20]. This does not indicate a lack of power to estimate genetic effects; rather, the variance of off‑diagonal relationships was sufficient to estimate additive genetic variance, as reflected by the standard errors of our estimated genetic parameters.

Following confirmation of no population stratification, variance components and heritability estimates were obtained using LMMs. The variance components estimates were all significantly different from zero across traits (Table 4). This suggests that the tracking methodology was sufficiently informative to allow the models to capture the genetic component of the traits derived from the data. The heritability estimates based on hourly records were low for all traits because of the higher impact of the environmental effects on observations obtained from shorter periods of time. This was evidenced by the heritabilities of daily measurements, which were approximately three times higher than those from hourly measurements (Table 5). This aligns with theoretical expectations: averaging repeated measures reduces the impact of transient environmental effects and residual variance, thereby increasing the proportion of variance attributable to genetic differences.

Repeatability is a fundamental parameter for estimating genetic effects when multiple measurements are obtained from the same individual, as it represents the proportion of phenotypic variance attributable to both genetic and permanent environmental effects. The repeatability values observed across traits (Table 4) indicate that our tracking methodology successfully detected individual-dependent patterns of behaviour. Since heritability cannot exceed repeatability, these values define the maximum proportion of variance that could be explained by genetics [30]. In our case, the estimated variance for the permanent environmental effect was at least twice as large as the genetic variance, which shows that much of the behavioural consistency we observed in the hourly traits is non-genetic. Nonetheless, a significant genetic component was still clearly estimable.

Although 01_DH was a binary trait, we chose to analyse it using LMMs rather than threshold models. This decision was supported by previous studies showing that LMMs can provide a good approximation of threshold models when the population average of a binary trait is not close to either 0 or 1 [34]. Moreover, EBVs derived from linear and threshold models for categorical traits are typically highly correlated [35]. In our dataset, the phenotypic mean for 01_DH was 0.6 (Table 4), satisfying the conditions under which LMMs are considered reliable for binary data. Additionally, a correlation analysis between EBVs obtained from our LMM and those from a Generalized Linear Mixed Model (GLMM) confirmed that both were highly similar (*r* = 0.98, data not shown). Given the simplicity and interpretability of LMMs, and the negligible difference in outcomes compared to GLMMs in this context, we proceeded with LMMs for the analysis of all traits.

Unlike heritability, which expresses genetic variance as a proportion of the total phenotypic variance, the GCV scales the additive genetic standard deviation to the trait mean, providing a mean-standardized measure of genetic variability [36]. This allows the GCV to reflect the potential for genetic improvement of a trait, relative to its mean, through selection independently of environmental variation in the data (e.g., noise associated with hourly behavioural measurements). In this study, the GCVs were moderate across traits, and are comparable to those of established livestock production traits known to respond to selection. For example, based on the weighted means and weighted mean genetic variances reported in [37], we calculated GCVs for milk yield (~ 0.09), milk fat yield (~ 0.08), and milk protein yield (~ 0.08) in Holstein cattle that are of similar magnitude to those presented in Table 5. This comparison suggests that the potential for selection response in the studied traits is not unusually constrained when considered in the context of typical livestock breeding programs.

The initial rationale behind including both traits 01_DH and MDH was to capture related aspects of presence in the litter area. The aim of measuring both was to evaluate whether continuous detection data (MDH) provided additional information beyond the simpler binary measure of 01_DH, or if it merely introduced noise. At the genetic level, a strong positive genetic correlation was found (Table 6), confirming that these traits captured similar underlying genetic factors. However, the correlation being less than one also indicates that MDH offers additional information.

The strong negative genetic correlation between MDH and WSH was unexpected. While initially both traits were thought to capture aspects of activity in the litter area, this result suggests that hens are genetically predisposed toward one of two mutually exclusive behavioural patterns: either high litter occupancy or high movement. However, technical artifacts cannot be entirely ruled out. For instance, individuals with higher WSH may have moved quickly out of the camera’s field of view, resulting in shorter detection periods (lower MDH). This could create an artefactual inverse relationship between movement and detection time, rather than reflecting a true biological correlation. If the correlation reflects a true biological relationship, these results may suggest that individuals genetically predisposed to higher WSH might engage more in movement between system levels, aligning with evidence that hens display individual patterns of spatial movement [38]. Conversely, birds with higher MDH might be those spending longer periods in the litter for activities like standing or dustbathing, behaviours typically associated with less movement. These findings highlight the importance of validating behavioural phenotypes derived from tracking data, ideally for a variety of housing systems.

The moderately high model-based EBV accuracies across all traits (Table 5) are encouraging, suggesting that the tracking methodology successfully captures genetic variation among individuals, likely due to the large number of repeated observations per individual (Table 1) and the genomic relationships among individuals. Interestingly, the reported accuracies of EBVMP were substantially higher than the cross-validated ones, by roughly a factor of two (Table 7), which may reflect the limitations of theoretical reliability formulas in small datasets and for low-heritability traits [39]. Model-based methods assume perfect knowledge of genetic parameters and ignore environmental covariances, whereas cross-validation accounts for these uncertainties; consequently, model-based accuracies tend to overestimate true predictive performance, particularly in small or noisy datasets.

To estimate cross-validation accuracy, we avoided pre-correcting phenotypes for systematic or random effects and instead fitted LMMs regressing phenotypes on EBVMP while simultaneously accounting for systematic and random effects. The regression slopes did not indicate systematic over- or under-dispersion, suggesting that EBVMP were unbiased estimators of genetic merit despite their modest predictive accuracy. However, it is important to note that the large standard errors of these estimates mean that we cannot definitively confirm unbiasedness. Furthermore, the trivariate model failed to yield meaningful improvements in cross-validation accuracy compared to univariate analyses, despite moderate genetic correlations between some traits (e.g., between 01_DH and MDH; Table 6). A bivariate analysis of MDH and WSH (i.e., excluding the binary trait and its missing‑data pattern) produced nearly identical genetic correlations and similar predictive accuracies (data not shown), indicating that the values obtained with the trivariate model are not artifacts originating from including 01_DH in the analysis. This contrasts with theoretical expectations that multi-trait models can improve prediction accuracy when traits share genetic architecture [40].

Our results demonstrate that the ArUco marker tracking system can be used to successfully generate behavioural phenotypes with a detectable genetic signal, enabling the estimation of breeding values for traits previously inaccessible for genetic studies. This provides a functional framework for integrating behavioural data into genetic evaluations. Although modest accuracies and some data loss likely contributed to noise, these limitations did not prevent the identification of significant genetic variation.

However, the current implementation requires substantial manual effort, which limits its practical scalability. Constructing the backpacks alone took several weeks of manual labour, and fitting them required a full day of work for twelve people. Daily backpack losses (roughly ten per day across all pens) further contributed to detection gaps. Future development should focus on simplifying the entire workflow, including more durable backpacks and automated data processing. Moreover, integrating the tracking data with complementary behavioural observations (e.g., social proximity, dust-bathing, foraging) could greatly improve trait interpretability. Overall, our findings confirm the potential of this approach as a viable tool for behavioural analysis, with clear opportunities for refinement and expansion in future research.

## Conclusions

This study shows that automated tracking using ArUco markers can effectively capture individual behavioural variation with an underlying genetic component, enabling the estimation of breeding values for previously inaccessible traits. Despite technical challenges that led to data loss and modest predictive accuracies, this methodology proved sufficiently robust and powerful to significantly detect genetic variance without major bias.

However, the current implementation is hindered by high manual effort, limiting its immediate practicality. Future work must focus on simplifying the entire workflow, from data collection to analysis, to enhance scalability. When refined, this approach provides a scalable framework for integrating detailed behavioural phenotypes into genetic evaluations.

## Supplementary Information

Below is the link to the electronic supplementary material.

Additional file 1.

## Data Availability

The data is available upon request.
