# Supplementary material for: From pixels to breeding values: genetic analysis of detection and movement traits in laying hens using automated tracking data of ArUco-marked birds
Source: Genet Sel Evol. 2026 Apr 10;58:28. doi: 10.1186/s12711-026-01043-y (PMC13217794; doi:10.1186/s12711-026-01043-y)
Supplement: Supplementary file 1 — Additional file 1. [file 12711_2026_1043_MOESM1_ESM.docx]

**Additional file 1**

**Figure S1** Distribution of the individual-level average number of detection losses per detected hour in the focal zone. Description: Detection loss was widespread across individuals, although some individuals exhibited higher average loss rates.

**
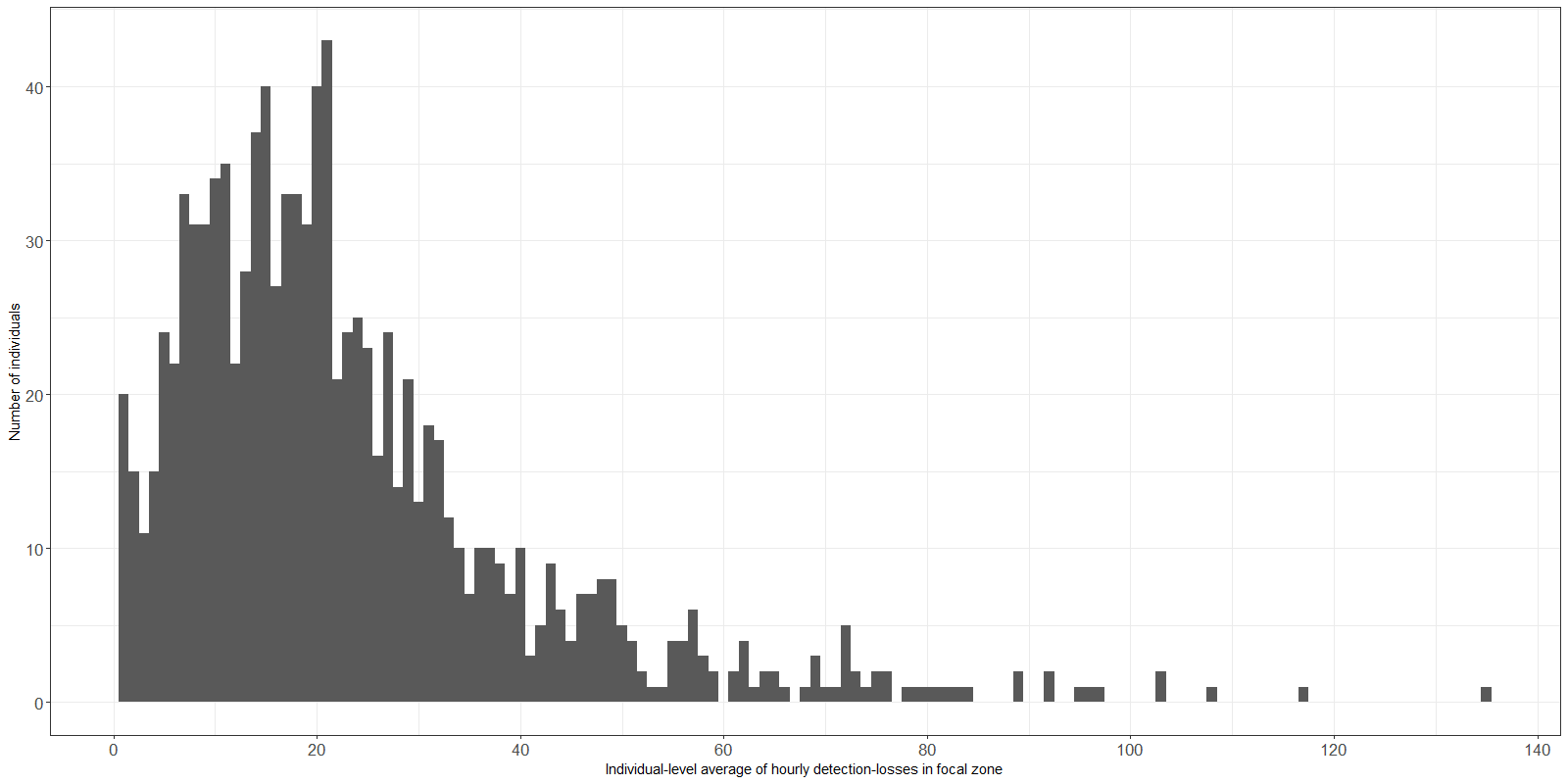
**

Table S1 Variance components across traits from trivariate model

| Trait | $\sigma_{a}^{2}$ (SE) | $\sigma_{c}^{2}$ (SE) | $\sigma_{d}^{2}$ (SE) | $\sigma_{e}^{2}$ (SE) |
| --- | --- | --- | --- | --- |
| 01_DH | 0.01  (± 0.004) | 0.07  (± 0.0005) | 0.05  (± 0.003) | 0.16  (± 0.0009) |
| MDH | 0.09  (± 0.03) | 0.06  (± 0.005) | 0.27  (± 0.02) | 0.86  (± 0.006) |
| WSH | 0.65  (± 0.24) | 0.54  (± 0.04) | 2.25  (± 0.18) | 8.31  (± 0.063) |

01_DH: detected or not per hour; MDH minutes detected per hour; WSH: walking speed per hour (in cm); $\sigma_{a}^{2}$ additive variance, $\sigma_{c}^{2}$ variance of pen-day-hour effect; $\sigma_{d}^{2}$ variance of permanent animal effect; $\sigma_{e}^{2}$ : residual variance.
